# Knowledge, perceptions, and practices around zoonotic diseases among actors in the livestock trade in the Lake Victoria crescent ecosystem in East Africa

**DOI:** 10.3389/fpubh.2023.1199664

**Published:** 2024-01-08

**Authors:** Hamilton Majiwa, Salome A. Bukachi, Dalmas Omia, Eric M. Fèvre

**Affiliations:** ^1^Institute of Anthropology Gender and African Studies, University of Nairobi, Nairobi, Kenya; ^2^International Livestock Research Institute, Nairobi, Kenya; ^3^Institute of Infection, Veterinary and Ecological Sciences, University of Liverpool, Neston, United Kingdom

**Keywords:** zoonotic diseases, knowledge, perceptions, practices, livestock trade

## Abstract

**Background:**

Zoonotic diseases such as anthrax, rabies, brucellosis, and Rift Valley fever pose a direct threat to health and undercut livelihoods in the communities in which they occur. A combination of anthropogenic and animal activities like migration and interaction with wildlife and their respective parasites and vectors drives the emergence and re-emergence of zoonotic diseases. Consequently, One Health interdisciplinary approaches that incorporate social scientists can provide key insights into complex local perceptions. The approach calls for collaboration between the human and animal health sectors, including the sharing of disease surveillance data necessary to alleviate disease impacts. Livestock traders interact closely with livestock, which puts them at elevated risk of infection and creates conditions by which they may spread zoonotic disease. It is thus essential to examine practices among actors involved in the livestock trade to understand the most appropriate ways to mitigate these risks.

**Methods:**

A qualitative study was conducted among the actors in the livestock trade in Busia County on their knowledge and perceptions of zoonotic diseases and practices that may contribute to the spread, control, and prevention of zoonotic disease transmission. A thematic analysis framework was used to categorize and synthesize data from in-depth interviews (IDIs), key informant interviews (KIIs), and structured observations.

**Results:**

Whereas participants could list livestock diseases, they could not identify which ones were zoonoses, demonstrating insufficient knowledge of zoonosis. They identify sick animals by checking for dropped ears, excess mucus production, diarrhea, bloody urinal discharge, and general animal activity levels. To prevent the spread of these diseases, they wash their animals, isolate sick animals from the rest of the stock, and vaccinate their animals. They seek help from animal health professionals for sick animals as part of curative practices. This shows that they perceive the diseases as serious and that they need to be attended to by professionals. The results also show that they perceive animals from outside the region to be more vulnerable to diseases compared to those from within. The actors in the livestock trade engage in practices like skinning dead animals before burying them; to them, this is a normal practice. Some also consume dead carcasses. These increase the risk of zoonotic disease transmission.

**Conclusion:**

The actors involved in the livestock trade are critical in the prevention and elimination of zoonotic diseases; hence, they need to be involved when developing intervention programs and policies for animal health extension services. Training them as a continuum of animal health workers blends lay and professional knowledge, which, alongside their intense contact with large numbers of animals, becomes a critical disease surveillance tool. Increasing awareness of zoonoses by using multi-disciplinary teams with social scientists is urgently needed so that practices like skinning dead animals before disposing of them and consumption of dead carcasses can be minimized.

## Introduction

Any infectious disease potentially transmissible from animals, both wild and domestic, to humans is defined as a zoonotic disease (1). The diseases that infect humans from animals are caused by bacterial, viral, parasitic, or fungal pathogens and spread to humans through bites, scratches, vectors, or ingestion. Zoonotic diseases are categorized according to their route of transmission, namely vector-borne like Rift Valley fever or food-borne such as *Campylobacter*, *Salmonella*, *E. coli*, and *Listeria*; pathogen types such as microparasites, viruses, bacteria, protozoa, worms, ticks, or fleas; or degree of person-to-person transmissibility such as coronaviruses (CoV) and Ebola virus (2–5).

Humans live in close relationships with domesticated animals. These animals may have pathogens that are transmissible to humans and can be harmful to health (6). Zoonotic diseases pose problems for global health; they account for an estimated 60% of known infectious diseases and 75% of emerging infectious diseases that are reported globally (7). Endemic zoonotic diseases are prevalent in developing regions worldwide, particularly in areas where humans and animals reside in close proximity. Endemic zoonotic diseases persistently impose a significant disease burden, particularly across tropical regions. They affect human health and wellbeing directly as common causes of human disease and indirectly through impacts on livelihoods and food security because of livestock production losses. Despite these multiple impacts, endemic zoonoses are still rarely recognized and are poorly understood (8). These diseases pose health burdens in addition to having negative social and economic effects on communities. The infected individual becomes unproductive, and close relatives spend money providing care and treatment. Time and money spent searching for a cure may put a severe drain on family resources (9). Zoonotic infections in people and animals occur in the context of a wide range of co-endemic pathogens in a rural community in western Kenya (10). The varying public health burden and socio-economic impact of zoonotic diseases across time and geographical settings make prioritizing their prevention and control important (11). The emergence of zoonoses, both recent and historical, can be considered a logical consequence of both pathogen and human ecology and pathogen evolution as microbes exploit new niches and adapt to new hosts (1). Access to these new niches is mediated by human action in most cases, including changes in land use, extraction of natural resources, animal production systems, modern transportation, antimicrobial drug use, and global trade (1).

The emergence of infectious diseases is a result of a variety of interconnected factors. These factors encompass population growth, alterations in dietary, farming, and trade methods, as well as changes in land use, such as rapid urbanization, deforestation, and encroachment on wildlife habitats. Furthermore, ancient zoonotic diseases are resurgent, such as rabies, anthrax, brucellosis, bovine tuberculosis, zoonotic trypanosomiasis, and conditions associated with tapeworm infections (12). This resurgence is attributed to a combination of similar factors, including the transmission of pathogens from wildlife to domestic animal populations (12). The appearance and re-appearance of many diseases, including zoonotic diseases, have been driven by the changing and increasing interconnection among humans and animals and the intensification of human activities surrounding and encroaching into natural habitats, enabling pathogens in wildlife reservoirs to spill over to livestock and humans over time. Cultural changes as a result of the rise in population, economic developments, technical developments, and intensification of farming have created more intense interaction between humans and livestock (13). The increased risk of disease emergence and the possibility of pervasiveness have been linked to increased regional trade and travel, an increase in human and livestock populations, and changing subsistence systems reflected in agricultural practices that have led to agricultural intensification and significant environmental changes in recent times (14). This warrants the need for continuous surveillance and disease monitoring and an understanding of the cultural epidemiology of zoonotic diseases.

People’s cultures, norms, knowledge, attitudes, and practices (KAP) can contribute to the spread, control, and elimination of various diseases, including zoonotic diseases (15), and also influence their health-seeking behavior. The KAP studies are popular as they help in assessing health-related beliefs and behaviors and how far community knowledge corresponds to biomedical concepts in the context of specific diseases and illnesses (16). Several KAP studies take a qualitative approach; however, qualitative KAP studies are also increasingly being carried out to get more in-depth perspectives on knowledge, attitudes, perceptions, and practices on health issues. Perceptions may be influenced by knowledge, cultural and religious practices, and lived experiences, and they in turn may predict action or behavior.

Social determinants of health are defined by the World Health Organization as conditions or circumstances in which people are born, grow, live, work, and age. These conditions are shaped by political, social, and economic forces (17). Apart from culture and norms, knowledge, perceptions, and attitudes—indicators of social inequality, including education and income—also play critical roles in determining access to healthcare as well as influencing how healthcare services are utilized (18). Kenya faces major health challenges that are influenced by various social and economic determinants such as access to safe water and adequate sanitation, nutrition, safe housing, occupational hazards, road safety, security, and income (19). Social determinants of health are shaped by public policies. The structure and quality of healthcare are considerably influenced by public policies made by governments (17). Social relationships impact adherence to medical treatment plans, seeking medical assistance, using healthcare services, and ultimately affecting health outcomes. Within healthcare organizations, social capital holds significance as it contributes to the effective provision of well-coordinated, high-quality care. These form very important concepts of social epidemiology (20, 21). Studies have shown that knowledge of reservoirs of zoonoses and how they are transmitted to humans has enabled early detection, reporting, and control (22). Knowledge or awareness about diseases varies across individuals and communities. Evidence shows that people’s perceptions about disease risks such as transmission and health consequences influence their attitudes and health-seeking actions and behaviors toward the diseases concerned (23). The One Health approach recognizes the interconnectedness of global health issues and, as such, promotes the importance of and need for international, interdisciplinary, and cross-sectoral communication and collaboration at local, national, and international levels (24). For One Health interventions to work, it is best to work closely with the local people and relevant disciplinary players to understand their local conditions and context (25). An interdisciplinary One Health approach that incorporates social scientists can provide insights into the complex local perceptions influenced by knowledge, religious beliefs, and lived experiences and how interventions can be designed or improved while acknowledging and addressing critical issues around awareness, perception, and underlying practices (26). The potential for enhancing public health outcomes through early detection of zoonotic disease events before they spread widely among humans lies in the collaboration between the human and animal health sectors. This collaboration encompasses the exchange of disease surveillance data, fostering a joint effort through the One Health approach to safeguard the wellbeing of humans, animals, and the environment (27).

The livestock trade system is a complicated chain with producers, traders, and numerous other market participants who are all referred to here as “actors in the livestock trade.” Animals travel from homes through different markets and trade routes to the last consumer or terminal market (28). Numerous informal chains with independent livestock and meat traders play a critical role in the livestock trade (29).

**Figure 1:**
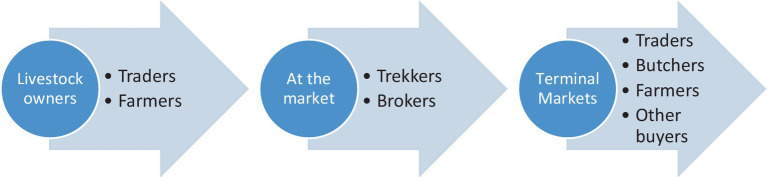
Different actors in the livestock trade.

The flow diagram (Figure 1) shows how various actors in the livestock trade are connected.

The actors in the livestock trade include the livestock keepers, who rear the livestock and make the decision to sell them. There are traders whose business is to buy and sell livestock. Brokers engage in connecting potential sellers to buyers and vice versa. There are also animal trekkers whose role is to walk the livestock from their homes to the markets or across markets. The livestock movement is an important driver of infectious disease transmission and spread (23). In Kenya, livestock movements are motivated by a need for animals to access resources (pasture and watering) to ensure their survival and when they are sold or being sold, as this study has revealed. Livestock often travel several kilometers each day to reach communal resource areas or markets where extensive mixing of herds and contacts between animals occur, with considerable implications for pathogen transmission and subsequent disease spread (30). There is evidence of cross-border trading of livestock in Busia, and given that the borders are highly porous to animal movement, this may contribute to zoonotic disease spread (31). The East African Community (EAC) partner states share a similar disease profile (32); their borders are also highly porous to informal animal and human movement, which is common in countries where animal production is not intensive (31). With the ease of livestock movement across borders, zoonotic diseases can spread across the countries, and therefore the Lake Victoria Crescent ecosystem, which has a dense human population, cross-border trade, and intensifying farming, was chosen for this study. Understanding livestock movement and having livestock movement data can facilitate disease control and surveillance (33). Lake Victoria Crescent forms the right environment for strategic zoonotic disease control programs through public health awareness campaigns among local and foreign livestock traders. The area is occupied by non-pastoralist communities, which depart from the usual focus on pastoralism when considering livestock movement. It forms a critical area in relation to livestock and zoonotic diseases. The study sought to bring out knowledge and practices on zoonotic diseases in non-pastoral areas and, more so, among actors in the livestock trade in the Lake Victoria Crescent ecosystem.

## Methods

### Study area

**Figure 2:**
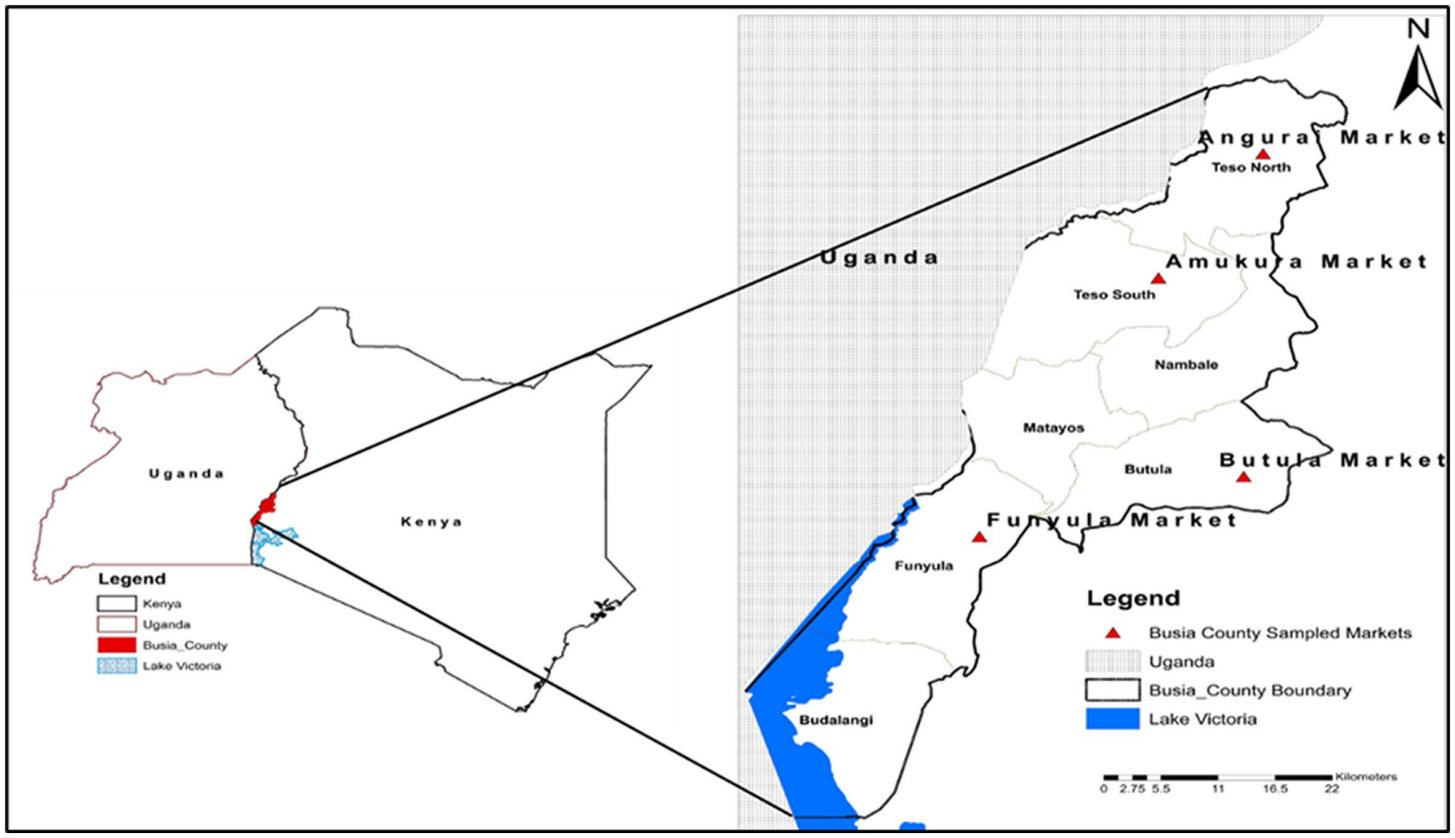
Map of the livestock markets in Busia County in the Western Kenya region.

The study was conducted in livestock markets in Busia County, Western Kenya, between November 2019 and March 2020. The markets were Amukura and Angurai, situated to the north; Butula, which was central; and Funyula, which is slightly to the south (Figure 2). Busia County is located in the western part of Kenya and broadly represents the Lake Victoria Crescent Ecosystem (10), where several zoonotic infections such as Brucellosis, Q-fever, bovine tuberculosis, human African trypanosomiasis (HAT), Rift Valley fever (RVF), and cysticercosis/taeniasis are co-endemic (10, 27). It borders Uganda with two border crossing points at Busia and Malaba towns. It is one of the four counties comprising the Western Kenya region and is situated at the extreme western border of the country.

### Research design

This exploratory study used in-depth interviews, key informant interviews, and unstructured observation to understand the knowledge and perceptions of zoonotic diseases and practices of the actors involved in the livestock trade. The study began with in-depth interviews (IDI) with livestock traders and trekkers on their knowledge, perceptions, and practices in relation to zoonotic diseases. Knowledge, attitudes, and practices (KAP) studies are commonly used to identify knowledge gaps and behavioral patterns among sociodemographic subgroups for the effective implementation of public health interventions (34). They provide useful information, are cost-effective, and are simpler to design and execute with limited time and budget as compared to a more in-depth ethnographic study (35). KAP surveys can identify knowledge gaps, cultural beliefs, or behavioral patterns that may facilitate understanding and action. They can identify information that is commonly known and perceptions or attitudes that are commonly held. They can help identify factors influencing behavior that are not known to most people, reasons for their attitudes or perceptions, and how and why people practice certain behaviors. One drawback associated with these types of research is the tendency to overlook other forms of knowledge, given the predominant emphasis on biomedical knowledge. Additionally, assessing attitudes remains a challenging task within such studies. Questions regarding practices often fail to encompass real-world behaviors or account for influential contextual elements, potentially impacting their credibility. Nevertheless, KAP studies can prove highly valuable when it comes to appraising communal understanding and gaging shifts in knowledge after interventions such as awareness-building through media initiatives and educational workshops (36).

The in-depth interviews were followed by key informant interviews (KII) with the market chairperson, chairperson of the traders, chairperson of the trekkers, officers in charge of animal health at the market, and the market masters, who are county officials in the market. The in-depth interviews teased out the actors’ knowledge and practices on zoonotic diseases; the key informant interviews then followed this. The key informant interviews aimed to provide more insight into the actors’ knowledge of the diseases, their perceptions of the diseases at the community level, and some of the practices that may contribute to spreading or controlling zoonotic diseases. All the interviews were audio-recorded with consent from the study participants. The audio data were transcribed once the qualitative data from the in-depth interviews and key informant interviews were obtained. Where the interviews were not conducted in English, the audio data were translated and transcribed. The transcription was done verbatim. Transcription involves converting recorded audio, typically spoken language, into a written format to analyze a specific occurrence. The process of transcription is known to be time-consuming and often monotonous, requiring several hours to complete. Responding to the demand for enhanced transparency and reproducibility in scientific methods, the creation of accurate, comprehensive, and systematically constructed transcripts in both the source and target languages plays a crucial role (37, 38). Thematic framework analysis, a method for systematically identifying, organizing, and offering insight into patterns of meaning (themes) across a dataset (39, 40) was used to systematically categorize and synthesize the qualitative data the interviews generated. All transcripts were read and reread for salient themes of how they identify sick animals, the common diseases that affect their livestock, and diseases that originate from animals to humans to respond to the assessment of knowledge and what action they take when they suspect an animal is sick, what preventive measures they take to avoid the spread of disease, and how they dispose of dead animals under their care to assess their practices. Under knowledge, the study looked for the respondents’ knowledge of the symptoms of zoonotic diseases, the causes, the mode of transmission, and the availability of treatment options. Under attitudes, the study analyzed how the respondents perceive and evaluate zoonotic diseases. Under the theme of the practice, the study analyzed the respondents’ conscious behaviors and actions toward sick animals. These actions could be informed by lay or acquired knowledge.

### Sample and sampling procedure

Purposive sampling was employed to identify 30 actors (both male and female) among the livestock traders (41). The traders and trekkers were identified with the help of their market chairpersons, who functioned as gatekeepers. The chairpersons held authority over the market and managed various matters. They oversee all market affairs, including dispute resolution. Positioned as an outsider, engaging with them was vital to fostering trust and explaining the purpose of my study to the prospective participants, ensuring they understood that it was academic research and not associated with any monetary incentives. Informants, who included animal health officers, market officials, and county government officials, were purposefully sampled for key informant interviews.

### Data collection methods

In-depth interviews (42) were conducted with 30 informants; the interviews lasted between 25 and 30 min. The interviews were conducted in the markets, away from crowds, to enable the informants to continue their operation without much interference. They were audio-recorded with consent from the study participants. Hamilton Majiwa, who is one of the authors and a native of western Kenya, conducted the interviews in Kiswahili. It was important to conduct the interviews in the markets to be able to observe the operations in the market in real time and to put into context the information given. In some instances, we experienced challenges from noise and curious onlookers. Curious onlookers were explained that only the responses of the selected respondents were needed at that time; they understood and walked away. Others expressed interest in participating, and we recruited them as participants in the study. This method allowed the respondents to express themselves in their own ways without restriction, as would happen with questionnaires (43). The interviews were audio-recorded with permission from the informants. Key informant interviews were also conducted with nine key informants. The interviews were also audio-recorded with permission from the informants. Unstructured observations (44, 45) were made in all the markets sampled by Hamilton Majiwa, who is one of the authors. The observations lasted between 30 and 45 min. Observation notes were taken, and these helped in understanding the activities in the market, like when the traders arrive, what other activities go on in and around the market, and how the actors in the livestock trade interact and conduct their operations within the market.

### Ethical statement

Participants gave informed written consent before participating in the study. The study was approved by the International Livestock Research Institute Institutional Research Ethics Committee in Kenya (ILRI-IREC2017-08), which is registered and accredited by the National Commission for Science, Technology, and Innovation in Kenya, and approved by the Federalwide Assurance for the Protection of Human Subjects in the United States. A research permit was obtained from the National Commission for Science, Technology, and Innovation (NACOSTI) under License No. NACOSTI/P/19/2547.

## Results

This study comprised 30 informants with distinct roles interviewed using in-depth interviews, key informant interviews, and observations. The informants consisted of 15 traders and 15 trekkers. Table 1 shows the profiles of the study participants.

**Table 1:** Demographic profile of the informants.

Characteristic	Frequency (*n* = 30)
*Sex*	
Male	29
Female	1
*Age in years*	
18–29	11
30–39	9
40 and above	10
*Education level (Kenyan education system)*	
With primary education	13
With secondary education	12
Above secondary education	5
*Religion*	
Christianity	29
Islam	1
*Ethnicity*	
Teso	11
Samia	13
Luo	6
*Years in the livestock trade*	
1–5	6
6–10	12
11 and above	12

### Profile of the respondents

The study found that of the 30 informants, only 5 had completed secondary education. Most of the traders were over 30 years old, with the oldest being 60 years old, while the trekkers’ ages ranged between 18 and 50 years old. In contrast to the trekkers, who were all male, among the traders, there were three females. All 15 trekkers and 14 traders identified themselves as Christians, while only one trader identified themselves as Muslim. Religion formed an important demographic characteristic because of certain food behaviors and practices that are influenced by religion.

Similar to a previous study (46), in this study, age, education level, religion, and sex did not seem to influence actors’ knowledge and awareness toward zoonotic diseases, as the responses given did not differ much depending on these variables. However, religious affiliation affected practices as some informants reported that they do not eat animals that have not been slaughtered because this goes against their religious beliefs. Similarly, those who were of the Muslim faith reported that they do not interact with or deal with pigs in any way. Ethnicity played a crucial role in livestock trading relationships. The study observed the presence of several ethnic-based clusters in the sampled markets. Traders from the same area or ethnic community tended to congregate in specific sections of the markets. This made it easier to identify traders and animals from different regions, such as Nandi County, which is a county located in the north Rift Valley in Kenya, or even those from neighboring Uganda. The ethnic composition of the informants was diverse, including individuals from the Teso, Samia, and Luo ethnic groups.

Religion, as one of the social demographics in this study, has a significant relationship to the practices that the actors engage in. Religious beliefs could potentially influence perceptions, actions, and subsequent One Health outcomes. Religion and religious rituals have been associated with infections and infectious diseases such as RVF, Ebola, and COVID-19 (47). Religion emerged as a key factor influencing the practices of the actors involved in the study. It played a role in shaping the informants’ behaviors and perspectives toward livestock trading and zoonotic diseases. Muslim informants do not trade in pigs or pork-related products.

The study found that many traders are introduced to the trade through an apprenticeship by their relatives or friends. As a channel for achieving social inclusion, an apprenticeship offers opportunities and avenues to develop skills and proficiency in a trade (48). Apprenticeship, the process of developing from novice to proficient under the guidance of a skilled expert, varies across cultures and among different skilled communities; in many instances, it offers an ideal point of entry, and this was evident from the responses from the study participants like the one below.

“*Before you start this job, you must have someone who is helping you because you cannot know what size of cow is sold for what price. You must have someone to guide you on the prices.*” (IDI)

Starting the practice for trekkers seems to require low capital investment in terms of financial capital but more in terms of social capital; they need to be trusted, dependable, and popular to get business from the traders, as reflected in these excerpts:

"*You need to be known to the traders and the buyers and to be trusted because someone cannot give you their animal if they don't trust you and know where you come from."* (IDI)

*"For you to start as a trekker, you don't need capital; you just need to be known and trusted and also have an identity card."* (IDI)

The findings show that formal training was not a key component in terms of being a trader or a trekker. A key informant at Butula Market indicated,

*"There is no training that one undergoes; they just come and start the business.*" (KII)

The findings also indicate that information on how to identify sick animals used to be communicated to the traders, but that no longer happens. A key informant at Amukura Market responded to a question on whether the traders and trekkers undergo any training.

"*We used to call them in the market and give them some basic education on how to identify sick animals, but we don't do that anymore. Now we just walk and inspect animals and remove the sick ones from the market.*" (KII)

"*I do not know if they undergo any training, but what I know is that anybody can just come and join the trade provided he or she has capital.*" (KII)

In terms of learning the trade, apprenticeship on approximation and negotiation for prices, selection of good animals, and identification of sick animals were mentioned as very crucial by all the informants because they are what traders need to succeed in the business of livestock trade.

"*Before you start this job, you must have someone who is helping you because you cannot know what size of cow is sold for what price. You must have someone to guide you on the prices.*" (IDI)

"*There is no formal training; however, people who have experience in the business must teach you how to negotiate prices. The experienced traders also teach you how to select a good animal that will give you good returns.*" (IDI)

## Knowledge

The study sought to bring out the actors’ knowledge of zoonotic diseases. Therefore, the questions explored if the informants knew about zoonotic diseases and if they could name some of the zoonotic diseases, their modes of transmission, and some of the symptoms of zoonotic diseases. By asking the participants how they acquired knowledge of livestock diseases, it was established that vernacular radio stations, seminars, and workshops, through which information on livestock diseases is disseminated, have contributed to their knowledge of livestock diseases. The radio stations air programs that teach about livestock diseases. Seminars and workshops organized by the Busia County government Department of Veterinary Services and other non-governmental organizations working in the region also teach them about livestock diseases.

### Knowledge of zoonotic diseases

The findings show that some informants know that diseases can come from animals and infect humans. We note that both the livestock traders and trekkers have some knowledge of zoonotic diseases, as demonstrated by the fact that most respondents know that there are livestock diseases; however, not all of them know that diseases that affect humans can originate from animals. The traders showed more knowledge of zoonotic diseases compared to the trekkers. Many of the informants named specific diseases such as foot and mouth disease (FMD), brucellosis, lumpy skin disease (LSD), and anthrax. This could be because they are the most common diseases in the area, as was corroborated by the key informant as shown below.

*"The most common diseases in this area are Lumpy skin disease, foot and mouth, black water and anthrax although we have not had an outbreak recently in this market."* (KII)

The actors in the livestock trade know the clinical signs of livestock diseases in general. They have ways of identifying sick animals in general, and these do not necessarily indicate a zoonotic disease infection. The actors in the livestock trade identify sick animals by checking if they have: dropping ears, a lot of mucus in the nose, diarrhea, blood-stained urine, and low activity levels. This knowledge is shared by both traders and trekkers.

The results revealed knowledge of the transmission of zoonotic diseases through the consumption of meat and milk from infected animals; this was the most common among the respondents.

*"I know diseases that can come from animals to humans, such diseases can infect you if you eat meat from infected animals.”* (IDI)

From the study, the level of knowledge varies among the different actors; some can name some of the diseases and the modes of transmission and even identify the clinical signs, while others do not know of any diseases that can be transmitted from animals to humans.

### Perception of zoonotic diseases

Perception toward zoonotic diseases can be influenced by various factors, such as cultural beliefs, religion, personal experiences, and access to information. These perceptions may evolve as new outbreaks occur, scientific understanding advances, and public health measures are implemented. The study sought to assess the perceptions of the different actors in the livestock trade toward zoonotic diseases. Actors in the livestock trade with a positive perception toward zoonotic diseases can contribute significantly to disease prevention and control. These individuals prioritize the health and welfare of both animals and humans. They understand the potential risks associated with zoonotic diseases and take proactive measures to minimize their transmission. On the other hand, negative perceptions among actors in the livestock trade can exacerbate the spread of zoonotic diseases. Actors with negative perceptions and careless or complacent attitudes may overlook the potential risks associated with zoonotic diseases, leading to inadequate disease control measures. This negligence could result from a lack of awareness. Some of the responses received from the informants are below.

*“I have seen diseases in cows, mostly cows from Uganda. We have cows from Uganda, but when sold here in Kenya, they die easily from diseases.”* (IDI)

*“The animals from Uganda are the ones with diseases, their meat is reddish, tasteless, and very light, The Kenyan Government should put measures in place to first screen animals from Uganda for diseases at the border before they are allowed in Kenya. This will help in the control of diseases.”* (IDI)

The study revealed that the actors perceive that most of the livestock diseases and zoonotic diseases originate from outside Busia County, and most are brought in by animals from Uganda. They also perceive the animals from Uganda to be more vulnerable to diseases as compared to the ones from Kenya. The animals from Uganda are therefore sold to butchers for meat and not to farmers for breeding. This could be a considerable risk if indeed the animals have diseases because it will expose consumers to the risk of infection.

On the issue of where they would get help from in case of a zoonotic disease infection, most informants responded that they would seek help from a hospital. The study also found out that most informants believe in conventional medicine and that there are healthcare facilities in many parts of the study area that are easily accessible to the residents of the study sites. Below are some of the responses received from the informants.

*“In case of a suspected zoonotic disease infection, I will get help from the hospital, we have hospitals close by. We have Bumala and Murumba hospitals.”* (IDI)

*“If I get infected, I will get help from a hospital. The nearest hospital is about two kilometers from my home in Malakisi.”* (IDI)

The study found that most of the actors will get help from a hospital in case of an infection or suspected infection with a zoonotic disease. They will call a veterinary doctor to help their animals. This was the response from many of the actors interviewed. This shows that they perceive zoonotic diseases as serious infections that need professional attention.

## Practices of actors in the livestock trade

The actors have the practice of moving animals from one market to another, and some of the animals are moved into Kenyan markets from Uganda and vice versa. Results from the IDIs in the various markets indicate that up to five different markets are visited by the traders in a week and up to 20 in a month, trekking the animals from one market to the next. This can contribute to the spreading of diseases if an infected animal is moved from one market to another.

*“I buy animals from different markets even from markets like Bukedia in Uganda. I go to different markets like Angurai, Myanga, Bungoma, Amukura, and even Nambale. The animals are trekked to different markets.”* (IDI)

*“I go to Amukura, Nambala, Kemodo, Myanga and we have a new one called Segero in Teso south. I go to the market every day of the week apart from Sunday.”* (IDI)

There are those markets that the traders prefer going to more than others because of better prices and demand for the livestock.

There are also practices such as culling sick animals for consumption and the consumption of dead animals that have been shown to influence the transmission of zoonotic diseases (49). This kind of practice presents a risk of exposure to pathogens as well as the spread of pathogens to many other members of the community, contributing to the possibility of zoonotic disease infection in people. The study was informed of some practices that the actors engage in that are embedded in their cultural and religious beliefs. They engage in these practices because they believe that there is influence from a higher power that they cannot challenge or question. Most of these practices revolve around the way they would dispose of a dead animal. A question was asked about what the actors would do if an animal under their care died.

*“In our tradition, most people don't like throwing meat away because you will throw away your luck, so even if you don't want to eat it, you call people who want and give it out."* (IDI)

*“Some people say that if you bury a cow you have thrown away your luck and therefore a dead cow should be eaten, but some of us who are born again believe that luck comes from God when a cow dies, we bury it so that we can be safe from other diseases.”* (IDI)

The study identified circumstances where some actors will eat meat from an animal that has died from an unknown cause because they believe that throwing away meat will bring bad luck to them. The study also shows a widespread practice among the actors to skin their animals when they die before burying them. This practice was mentioned by most of the respondents in different markets, and the reason for doing this was reported to relate to cultural beliefs among the informants from Teso, Samia, and Luo communities. They believe that if one does not do this, they will never be successful in the business, as burying an animal with the skin is akin to burying all one’s wealth and luck. These practices seemed to cut across the three main ethnic groupings in the region.

Some practices were reported to be used by the actors in the livestock trade to control the spread of zoonotic diseases. The study findings indicate that there are practices that the actors in the livestock trade have adopted that help in the prevention of the spread of livestock diseases and, to an extent, the spread of zoonotic diseases. This involves activities such as the isolation of sick animals and separating animals that have come from one market and are bound to another market. Furthermore, the market chairpersons have also instituted rules that animals exhibiting any signs of sickness are not allowed in the ring where the animals are traded. In some markets, there are isolation crèches/structures for animals diagnosed at the market gates. The ones that find their way in are removed, and the owners are told to get help and only return them when they are well. This practice is enforced by the market chairperson and his team, “youths.” The traders are also vigilant and will report any animal that appears sick in the livestock ring.

*“I isolate sick animals, and then I call a veterinary doctor to come and help by treating it. I also ensure that dead animals are buried.”* (IDI)

*“If I am moving many animals and one of them falls sick, isolate it and tie it in a nearby homestead. I then inform the owner of the animal, who will then take action by calling a doctor. Then, I continue with the rest of the animals to my destination.”* (IDI)

The practice of isolating sick animals was reiterated and confirmed by key informants.

*“When we identify a sick animal in the market, we remove it from the market and advise the owner to get treatment for it and only bring it to the market when it is healed.”* (KII)

The actors in the livestock trade seek help from animal health professionals when they identify that their animal is sick. They also identified the uptake of animal vaccination to prevent infection and the spread of diseases. These practices show that they know disease transmission and prevention. The study found that vaccination exercises are done when there is an outbreak, and these are normally government initiatives to prevent the further spread of the disease. There is also the issuance of movement permits by the veterinary office for animals that are being moved from one county to another.

*“We issue movement permits to control diseases. Animals from an area with notifiable diseases will not be issued with movement permits.”* (KII)

## Discussion

As with other similar studies on knowledge of zoonotic diseases, the study revealed that the actors in the livestock trade in Busia do not have detailed knowledge of specific zoonotic diseases or details of disease control. Studies in Sub-Saharan Africa have pointed to limited knowledge among various actors on zoonotic diseases (23, 50). Many of the informants interviewed in this study know about livestock diseases but not much about diseases that can be transmitted from animals to humans and vice versa. Some informants mentioned brucellosis as a disease that can be transmitted from animals to human beings, which shows that some informants know brucellosis as a zoonotic disease. This is consistent with a study by Seyoum et al. (51) on knowledge, attitude, and practice among small-scale dairy farmers on milk-borne zoonotic diseases. Many study participants know about the potential health risks of drinking raw milk and link this practice to brucellosis. Anthrax, foot and mouth disease, and lumpy skin diseases were the diseases that most of the informants mentioned as the most common, and this was also affirmed by the key informants, although there was no recent outbreak of cases reported in the area at the time of the study. The fact that most of the actors mentioned these diseases could be attributed to the fact that whenever there is an outbreak, livestock markets are closed and quarantine of livestock is enforced.

The study found that the actors take zoonotic diseases seriously, even though a majority do not know about the existence of infections that can come from animals to humans. It is consistent with a study by Abdi et al. (50) that found a positive attitude toward zoonotic diseases; a high percentage of respondents had a positive attitude toward the prevention and control of RVF, a zoonotic disease. The positive perception is shown by the fact that they have preventive measures that they take, such as washing animals to remove ticks, taking animals for vaccinations, and visiting hospitals in cases of suspected zoonotic disease infection. The study also revealed that some actors believe that meat should be cooked appropriately to prevent zoonotic disease infections and that milk should not be consumed raw. The actors in the livestock trade take several measures to prevent the spread of zoonotic diseases and the occurrence of livestock diseases. The study also revealed that many actors seek help from hospitals in cases of zoonotic disease infection and from a veterinary doctor in cases where their animal is sick. This shows that they perceive the infections as serious. Recognizing the potential seriousness of diseases that can be transmitted between animals and humans, these actors in the livestock trade understand the importance of prompt medical intervention. By turning to a hospital, they aim to receive the necessary expertise to accurately diagnose, effectively treat, and prevent the further spread of such illnesses. By prioritizing their health and the wellbeing of their animals, actors demonstrate a proactive approach to managing zoonotic diseases to safeguard both themselves and their animal companions.

The actors’ reliance on hospitals and veterinary doctors reflects a broader understanding of the interconnectedness between human and animal health. Their actions underscore the importance of the One Health collaborative efforts among medical professionals and veterinarians to effectively address zoonotic diseases and mitigate their impact. However, the study also revealed some negative perceptions among some actors in the livestock trade who believe that the animals that originate from outside Busia County and especially from Uganda are the ones that have diseases and are even calling for the restriction of such animals. This is consistent with a study by (52) that found negative attitudes toward zoonotic diseases in bush meat hunters and traders in Nsukka, southeast Nigeria.

The traders also perceive the animals from Uganda to be more vulnerable to diseases as compared to the ones from Kenya. The animals from Uganda are therefore sold to butchers for meat and not to farmers for breeding. This could be a considerable risk if the animals indeed have diseases because it will expose consumers to the risk of infection. The livestock traders do this to get a market for their animals and make a profit.

The study revealed that the actors have ways of identifying sick animals. These are not necessarily animals suffering from zoonotic infections, but just ways of knowing if an animal is sick. This is consistent with the findings of Onono et al. (53). These ways of identifying sick animals were noted as familiar to both trekkers and traders, and they included dropped ears, excess mucus production, diarrhea, bloody urinal discharge, and general animal activity levels. This knowledge of identifying sick animals was found to be passed from one person to another and is not acquired through formal training.

One of the drivers of One Health is the association between humans and animals, and this association is influenced by religious beliefs in different communities. Studies have shown the importance of religious beliefs and practices in the perception of human health (54). Religious beliefs could potentially influence perceptions, actions, and subsequent One Health outcomes. Studies have also shown that practices such as herding, residing with livestock, slaughtering, skinning, and consuming meat and milk from ill or dead livestock play a vital role in transmitting zoonotic diseases like Rift Valley fever (RVF) to humans (55, 56). Cultural practices like traditional African burials during the Ebola outbreak in the Democratic Republic of Congo and Sierra Leone and the reliance on traditional healers for treatment of malaria in Côte d’Ivoire have been shown to play a significant role in disease outbreaks (57, 58). Different ethnic groups and those from different religious backgrounds have varied beliefs and practices, as shown in the findings where the Samia and Teso do not believe in throwing away meat or burying dead animals without skinning them. Muslims do not come into contact with pigs in any way. This study found that some actors believe that when an animal dies, it must be skinned before it is disposed of. They also believe that meat cannot be thrown away, and therefore, when an animal dies, the meat will be eaten or given to people who want to eat it. Such practices as skinning dead animals before burying them and eating meat from dead animals are based on the culturally embedded beliefs of the Teso, Samia, and Luo, who believe that if they do not adhere to them, bad luck will follow them, and they will not prosper in the business of livestock trade or rearing livestock. Such practices significantly expose them to the risk of infection. This is consistent with a study (59) that found that pastoralists engage in practices embedded in their culture that expose them to the risk of zoonotic disease infections.

Results from the study also showed that the actors have practices such as separating sick animals from the rest, not allowing sick animals in the market, and vaccinating their animals, which can prevent the animals from spreading diseases (60–62). Unlike other studies that have shown that pastoralists self-medicate their animals for different reasons, like having high confidence in their abilities and low confidence in the skills of animal health service providers, limited access to animal health service providers, and high cost (63–65) this study found that the livestock traders engage the help of those that they perceive to be animal health experts whenever they identify that their animal is sick.

There are globally relevant zoonoses that everyone worries about and locally relevant ones that are important, such as anthrax, trypanosomiasis/HAT, rabies, brucellosis, and RVF (11). These diseases can spread rapidly in a particular region (epidemics) or spread widely in many countries worldwide (pandemics), leading to massive losses of life and livelihoods and having a significant economic impact. Understanding a community’s knowledge and practices on zoonotic diseases is particularly important because it provides critical information to help design appropriate control and intervention measures for a zoonotic disease outbreak.

## Conclusion and recommendation

The study revealed a lack of knowledge on zoonoses among several of the traders and trekkers in the livestock trade that participated in the study. They were not aware of any diseases that are transmissible between animals and humans. Even though few of them know about zoonotic diseases, some of the actors’ practices show that they are aware of livestock diseases, and they take measures against them. As shown in other studies, low awareness and poor knowledge of zoonoses in pastoral and agro-pastoral communities are likely to increase the risk of contracting zoonoses (46). The findings indicate that skinning dead animals and eating carcasses from dead animals are common. It is also evident from the study that the actors (both traders and trekkers) move their livestock from one market to another and from one county to another, that is, Busia, Bungoma, and Kakamega, and some of them source animals from Uganda. This practice of the movement of livestock can contribute to the spread of zoonotic diseases from one region to another and across international borders. There is some control to ensure animals do not move from regions with notifiable diseases like foot and mouth disease or anthrax. This is in the form of the issuance of movement permits; however, the study noted that this was not effective as movement permits are only issued in some markets. The study found that of the markets under study, only one market had movement permits being issued, and it was only for animals that had been sold. There was no one checking if the animals coming to the market were from regions free of diseases. Even though several actors in the livestock trade interviewed do not know zoonotic diseases, they have in place practices that can help control these diseases, such as vaccination of animals, washing animals, separating animals for sale and those being kept at home, and ensuring that sick animals do not enter the market. These practices are likely to reduce the risk of animals getting infected and, in turn, infecting them with zoonotic diseases.

From the study findings, it is evident that cultural issues are important considerations in the control of infectious diseases, and therefore the study recommends incorporating cultural epidemiology and the One Health approach in the development of disease prevention, management, and control programs or interventions. Workers in the livestock sector need to be included in the development of interventions and policies for the control of zoonotic diseases so that their traditional beliefs and practices guided by experts in animal and human health are taken into consideration, without which some of the interventions may not work. The practice of skinning dead animals before disposing of them can be done more safely by having designated people do it since it is a firmly rooted cultural practice among the study participants. The study finds that the locals have a high propensity for information from vernacular radio stations, seminars, and workshops; therefore, it recommends the use of these for awareness creation on zoonotic diseases. It also recommends the use of cross-border intervention programs such as education and sensitization of the actors in the livestock trade in this Lake Victoria Crescent ecosystem and East Africa to help strengthen disease surveillance and control. In addition, the study also recommends the following:

1. Awareness creation of zoonotic diseases among the actors in the livestock trade.
2. Awareness creation and culture change programs targeting eliminating the practice of eating meat from sick animals or those that died of disease. This can be done by social scientists working together with public health officers and veterinary officers in the area.
3. The study also recommends further research: (1) exploring KAP on zoonotic diseases at household levels because animal production is at household levels and (2) exploring the community prioritization of zoonotic diseases and exploring KAP on specific zoonoses such as RVF, brucellosis, and anthrax as the study respondents mentioned them.

The findings of this study can be applied in similar settings, especially the cross-border points on control of zoonotic diseases.

## Data availability statement

The original contributions presented in the study are included in the article/supplementary material, further inquiries can be directed to the corresponding author.

## Ethics statement

The studies involving humans were approved by a research permit obtained from the National Commission for Science, Technology, and Innovation (NACOSTI) under License No. NACOSTI/P/19/2547. The studies were conducted in accordance with the local legislation and institutional requirements. The participants provided their written informed consent to participate in this study. The study was approved by the International Livestock Research Institute Institutional Research Ethics Committee in Kenya (ILRI-IREC2017-08), which is registered and accredited by the National Commission for Science, Technology, and Innovation in Kenya, and approved by the Federal wide Assurance for the Protection of Human Subjects in the USA.

## Author contributions

HM carried out the study; he developed the protocol and tools for the study and carried out the fieldwork, data collection analysis, and writing. SB is the supervisor, together with DO. They both assisted in reviewing the study and helping to improve it. They helped with reviewing the manuscript. EF won the grant for the study, and he also contributed to the review of the manuscript. All authors contributed to the article and approved the submitted version.

## References

[1] Karesh WB Dobson A Lloyd-Smith JO Lubroth J Dixon MA Bennett M. Ecology of zoonoses: natural and unnatural histories. Lancet. (2012) 380:1936–45. doi: 10.1016/S0140-6736(12)61678-X, PMID: 23200502 PMC7138068

[2] Lloyd-Smith JO George D Pepin KM Pitzer VE Pulliam JRC Dobson AP. Epidemic dynamics at the human-animal Interface. Science. (2009) 326:1362–7. doi: 10.1126/science.1177345, PMID: 19965751 PMC3891603

[3] Abebe E Gugsa G Ahmed M. Review on major food-borne zoonotic bacterial pathogens. J Trop Med. (2020) 2020:1–19. doi: 10.1155/2020/4674235, PMID: 32684938 PMC7341400

[4] Rahman MT Sobur MA Islam MS Ievy S Hossain MJ El Zowalaty ME. Zoonotic diseases: etiology, impact, and control. Microorganisms. (2020) 8:1405. doi: 10.3390/microorganisms8091405 32932606 PMC7563794

[5] Richard M Knauf S Lawrence P Mather AE Munster VJ Müller MA. Factors determining human-to-human transmissibility of zoonotic pathogens via contact. Curr Opin Virol. (2017) 22:7–12. doi: 10.1016/j.coviro.2016.11.004 27907884 PMC5346033

[6] Kayumba SM. Prioritization of zoonotic diseases in the Democratic Republic of the Congo, 2016. (2018).

[7] Salyer SJ Silver R Simone K Barton Behravesh C. Prioritizing Zoonoses for Global Health capacity building—themes from one health zoonotic disease workshops in 7 countries, 2014–2016. Emerg Infect Dis. (2017) 23:S55–64. doi: 10.3201/eid2313.170418, PMID: 29155664 PMC5711306

[8] Halliday JEB Allan KJ Ekwem D Cleaveland S Kazwala RR Crump JA. Endemic zoonoses in the tropics: a public health problem hiding in plain sight. Vet Rec. (2015) 176:220–5. doi: 10.1136/vr.h798, PMID: 25722334 PMC4350138

[9] Bukachi SA Wandibba S Nyamongo IK. The socio-economic burden of human African trypanosomiasis and the coping strategies of households in the South Western Kenya foci. PLoS Negl Trop Dis. (2017) 11:e0006002. doi: 10.1371/journal.pntd.0006002 29073144 PMC5675461

[10] Fèvre EM de Glanville WA Thomas LF Cook EAJ Kariuki S Wamae CN. An integrated study of human and animal infectious disease in the Lake Victoria crescent small-holder crop-livestock production system, Kenya. BMC Infect Dis. (2017) 17:457. doi: 10.1186/s12879-017-2559-6, PMID: 28666412 PMC5493856

[11] Munyua P Bitek A Osoro E Pieracci EG Muema J Mwatondo A. Prioritization of zoonotic diseases in Kenya, 2015. PLoS One. (2016) 11:e0161576. doi: 10.1371/journal.pone.0161576 27557120 PMC4996421

[12] Coker R Rushton J Mounier-Jack S Karimuribo E Lutumba P Kambarage D. Towards a conceptual framework to support one-health research for policy on emerging zoonoses. Lancet Infect Dis. (2011) 11:326–31. doi: 10.1016/S1473-3099(10)70312-1, PMID: 21376670 PMC7129889

[13] Chaiban C Robinson TP Fèvre EM Ogola J Akoko J Gilbert M. Early intensification of backyard poultry systems in the tropics: a case study. Animal. (2020) 14:2387–96. doi: 10.1017/S175173112000110X 32576312 PMC7538343

[14] Jones BA Grace D Kock R Alonso S Rushton J Said MY. Zoonosis emergence linked to agricultural intensification and environmental change. Proc Natl Acad Sci USA. (2013) 110:8399–04. doi: 10.1073/pnas.1208059110, PMID: 23671097 PMC3666729

[15] Kiffner C Latzer M Vise R Benson H Hammon E Kioko J. Comparative knowledge, attitudes, and practices regarding anthrax, brucellosis, and rabies in three districts of northern Tanzania. BMC Public Health. (2019) 19:1625. doi: 10.1186/s12889-019-7900-0, PMID: 31796011 PMC6889212

[16] Launiala A. How much can a KAP survey tell us about people's knowledge, attitudes and practices? Some observations from medical anthropology research on malaria in pregnancy in Malawi. AM. (2009) 11:13. doi: 10.22582/am.v11i1.31

[17] Islam MM. Social determinants of health and related inequalities: confusion and implications. Front Public Health. (2019) 7:11. doi: 10.3389/fpubh.2019.00011, PMID: 30800646 PMC6376855

[18] Artiga S Hinton E. Beyond health care: The role of social determinants in promoting health and health equity. (2018).

[19] Mauti J Gautier L De Neve JW Beiersmann C Tosun J Jahn A. Kenya’s health in all policies strategy: a policy analysis using Kingdon’s multiple streams. Health Res Policy Syst. (2019) 17:15. doi: 10.1186/s12961-019-0416-3, PMID: 30728042 PMC6366019

[20] Von Dem Knesebeck O. Concepts of social epidemiology in health services research. BMC Health Serv Res. (2015) 15:357. doi: 10.1186/s12913-015-1020-z, PMID: 26328943 PMC4557631

[21] Short SE Mollborn S. Social determinants and health behaviors: conceptual frames and empirical advances. Curr Opin Psychol. (2015) 5:78–84. doi: 10.1016/j.copsyc.2015.05.002, PMID: 26213711 PMC4511598

[22] Harper K Armelagos G. The changing disease-scape in the third epidemiological transition. Int J Environ Res Public Health. (2010) 7:675–97. doi: 10.3390/ijerph7020675 20616997 PMC2872288

[23] Swai ES Schoonman L Daborn C. Knowledge and attitude towards zoonoses among animal health workers and livestock keepers in Arusha and Tanga, Tanzania. Tanzan J Health Res. (2010) 12:272–7. doi: 10.4314/thrb.v12i4.54709 24409636

[24] Travis DA Chapman DW Craft ME Deen J Farnham MW Garcia C. One health: lessons learned from East Africa. Microbiol Spectr. (2014) 2:2.1.14. doi: 10.1128/microbiolspec.OH-0017-2012 26082115

[25] Gwakisa P George J Sindato C Ngonyoka A Nnko H Assenga J. Pillars for successful operationalization of one health as an ecosystem approach: experience from a human-animal interface in the Maasai steppe in Tanzania. One Health Outlook. (2023) 5:11. doi: 10.1186/s42522-023-00087-0, PMID: 37649116 PMC10469404

[26] Davis A Virhia J Bunga C Alkara S Cleaveland S Yoder J. “Using the same hand”: the complex local perceptions of integrated one health based interventions in East Africa. PLoS Negl Trop Dis. (2022) 16:e0010298. doi: 10.1371/journal.pntd.0010298, PMID: 35377878 PMC9009769

[27] Thomas LF Rushton J Bukachi SA Falzon LC Howland O Fèvre EM. Cross-sectoral zoonotic disease surveillance in Western Kenya: identifying drivers and barriers within a resource constrained setting. Front Vet Sci. (2021) 8:658454. doi: 10.3389/fvets.2021.658454, PMID: 34169106 PMC8217437

[28] Pavanello S. Livestock Marketing in Kenya-Ethiopia Border Areas (2010).

[29] Alarcon P Fèvre EM Murungi MK Muinde P Akoko J Dominguez-Salas P. Mapping of beef, sheep and goat food systems in Nairobi — a framework for policy making and the identification of structural vulnerabilities and deficiencies. Agric Syst. (2017) 152:1–17. doi: 10.1016/j.agsy.2016.12.005, PMID: 28260829 PMC5312657

[30] VanderWaal K Gilbertson M Okanga S Allan BF Craft ME. Seasonality and pathogen transmission in pastoral cattle contact networks. R Soc Open Sci. (2017) 4:170808. doi: 10.1098/rsos.170808, PMID: 29308225 PMC5749993

[31] Valerio VC Walther OJ Eilittä M Cissé B Muneepeerakul R Kiker GA. Network analysis of regional livestock trade in West Africa. PLoS One. (2020) 15:e0232681. doi: 10.1371/journal.pone.0232681, PMID: 32407336 PMC7224501

[32] Ope M Sonoiya S Kariuki J Mboera LEG Gandham RNV Schneidman M. Regional initiatives in support of surveillance in East Africa: the East Africa integrated disease surveillance network (EAIDSNet) experience. Emerg Health Threats J. (2013) 6:19948. doi: 10.3402/ehtj.v6i0.19948, PMID: 23362409 PMC3557906

[33] Chaters GL Johnson PCD Cleaveland S Crispell J De Glanville WA Doherty T. Analysing livestock network data for infectious disease control: an argument for routine data collection in emerging economies. Philos Trans R Soc B Biol Sci. (2019) 374:264. doi: 10.1098/rstb.2018.0264, PMID: 31104601 PMC6558568

[34] Lee M Kang BA You M. Knowledge, attitudes, and practices (KAP) toward COVID-19: a cross-sectional study in South Korea. BMC Public Health. (2021) 21:295. doi: 10.1186/s12889-021-10285-y, PMID: 33546644 PMC7863060

[35] Andrade C Menon V Ameen S Kumar PS. Designing and conducting knowledge, attitude, and practice surveys in psychiatry: practical guidance. Indian J Psychol Med. (2020) 42:478–81. doi: 10.1177/0253717620946111, PMID: 33414597 PMC7750837

[36] Bukachi SA Mumbo AA Alak ACD Sebit W Rumunu J Biéler S. Knowledge, attitudes and practices about human African trypanosomiasis and their implications in designing intervention strategies for Yei county, South Sudan. PLoS Negl Trop Dis. (2018) 12:e0006826. doi: 10.1371/journal.pntd.0006826, PMID: 30273342 PMC6181432

[37] Clark L Birkhead AS Fernandez C Egger MJ. A transcription and translation protocol for sensitive cross-cultural team research. Qual Health Res. (2017) 27:1751–64. doi: 10.1177/1049732317726761, PMID: 28936930 PMC5642906

[38] McMullin C. Transcription and qualitative methods: implications for third sector research. Volunt Int J Volunt Nonprofit Organ. (2023) 34:140–53. doi: 10.1007/s11266-021-00400-3, PMID: 34522070 PMC8432276

[39] Damayanthi S. Thematic analysis of interview data in the context of management controls research. London: SAGE Publications, Ltd. (2019).

[40] Nowell LS Norris JM White DE Moules NJ. Thematic analysis: striving to meet the trustworthiness criteria. Int J Qual Methods. (2017) 16:1773384. doi: 10.1177/1609406917733847

[41] Tongco MADC. Purposive sampling as a tool for informant selection. Ethnobot Res Appl. (2008) 5:147. doi: 10.17348/era.5.0.147-158

[42] Knott E Rao AH Summers K Teeger C. Interviews in the social sciences. Nat Rev Methods Primer. (2022) 2:73. doi: 10.1038/s43586-022-00150-6

[43] Jamshed S. Qualitative research method-interviewing and observation. J Basic Clin Pharm. (2014) 5:87–8. doi: 10.4103/0976-0105.141942, PMID: 25316987 PMC4194943

[44] Jibril A. Observational research in the social sciences: A neglected qualitative research technique (2014).

[45] Ekka PM. A review of observation method in data collection process, vol. 6 (2021). 12 p.

[46] Hundal JS Sodhi SS Gupta A Singh J Chahal US. Awareness, knowledge, and risks of zoonotic diseases among livestock farmers in Punjab. Vet World. (2016) 9:186–91. doi: 10.14202/vetworld.2016.186-191 27051206 PMC4819370

[47] Gajurel K Deresinski S. A review of infectious diseases associated with religious and nonreligious rituals. Interdiscip Perspect Infect Dis. (2021) 2021:1–9. doi: 10.1155/2021/1823957 PMC8668350 34912451

[48] Downey G Dalidowicz M Mason PH. Apprenticeship as method: embodied learning in ethnographic practice. Qual Res. (2015) 15:183–00. doi: 10.1177/1468794114543400

[49] Osbjer K Boqvist S Sokerya S Kannarath C San S Davun H. Household practices related to disease transmission between animals and humans in rural Cambodia. BMC Public Health. (2015) 15:476. doi: 10.1186/s12889-015-1811-5, PMID: 25952633 PMC4427931

[50] Abdi IH Affognon HD Wanjoya AK Onyango-Ouma W Sang R. Knowledge, attitudes and practices (KAP) on Rift Valley fever among pastoralist communities of Ijara District, north eastern Kenya. PLoS Negl Trop Dis. (2015) 9:e0004239. doi: 10.1371/journal.pntd.0004239, PMID: 26566218 PMC4643900

[51] Seyoum ET Mekonen TK Kebede N Gezahegn HA Mehirete TS Mengesha ZT. Knowledge, attitude and practice among small scale dairy farmers on Milk-borne zoonotic diseases, North Showa zone, ETHIOPIA (2016) 4:11.

[52] Ozioko KU Okoye CI Obiezue RN Agbu RA. Knowledge, attitudes, and behavioural risk factors regarding zoonotic infections among bushmeat hunters and traders in Nsukka, Southeast Nigeria. Epidemiol Health. (2018) 40:e2018025. doi: 10.4178/epih.e2018025, PMID: 29909609 PMC6178367

[53] Onono J Mutua P Kitala P Gathura P. Knowledge of pastoralists on livestock diseases and exposure assessment to brucellosis within rural and peri-urban areas in Kajiado, Kenya. F1000Research. (2019) 8:1916:1916. doi: 10.12688/f1000research.20573.1, PMID: 33204408 PMC7642991

[54] Vijayaraghavan G Tate V Gadre V Trivedy C. The role of religion in one health. Lessons from the Hanuman langur (*Semnopithecus entellus*) and other human–non-human primate interactions. Am J Primatol. (2022) 84:e23322. doi: 10.1002/ajp.23322, PMID: 34411317

[55] Bird BH Ksiazek TG Nichol ST MacLachlan NJ. Rift Valley fever virus. J Am Vet Med Assoc. (2009) 234:883–93. doi: 10.2460/javma.234.7.883 19335238

[56] Breiman RF Njenga MK Cleaveland S Sharif S Mbabu M King L. Lessons from the 2006–2007 Rift Valley fever outbreak in East Africa: implications for prevention of emerging infectious diseases. Future Virol. (2008) 3:411–7. doi: 10.2217/17460794.3.5.411

[57] Essé C Utzinger J Tschannen AB Raso G Pfeiffer C Granado S. Social and cultural aspects of “malaria” and its control in central Côte d’Ivoire. Malar J. (2008) 7:224. doi: 10.1186/1475-2875-7-224, PMID: 18973663 PMC2588631

[58] Lal A. Exclusivity of cultural practices within emerging disease outbreak responses in developing nations leads to detrimental outcomes. Front Public Health. (2021) 9:686540. doi: 10.3389/fpubh.2021.686540, PMID: 34295872 PMC8291361

[59] Mangesho PE Neselle MO Karimuribo ED Mlangwa JE Queenan K Mboera LEG. Exploring local knowledge and perceptions on zoonoses among pastoralists in northern and eastern Tanzania. PLoS Negl Trop Dis. (2017) 11:e0005345. doi: 10.1371/journal.pntd.0005345, PMID: 28146556 PMC5325590

[60] Carpenter A Waltenburg MA Hall A Kile J Killerby M Knust B. Vaccine preventable zoonotic diseases: challenges and opportunities for public health Progress. Vaccine. (2022) 10:993. doi: 10.3390/vaccines10070993, PMID: 35891157 PMC9319643

[61] Sander VA Sánchez López EF Mendoza Morales L Ramos Duarte VA Corigliano MG Clemente M. Use of veterinary vaccines for livestock as a strategy to control foodborne parasitic diseases. Front Cell Infect Microbiol. (2020) 10:288. doi: 10.3389/fcimb.2020.00288, PMID: 32670892 PMC7332557

[62] Msimang V Rostal MK Cordel C Machalaba C Tempia S Bagge W. Factors affecting the use of biosecurity measures for the protection of ruminant livestock and farm workers against infectious diseases in Central South Africa. Transbound Emerg Dis. (2022) 69:e1899–912. doi: 10.1111/tbed.14525, PMID: 35306739 PMC9790579

[63] Mangesho PE Caudell MA Mwakapeje ER Ole-Neselle M Kabali E Obonyo M. “We are doctors”: drivers of animal health practices among Maasai pastoralists and implications for antimicrobial use and antimicrobial resistance. Prev Vet Med. (2021) 188:105266. doi: 10.1016/j.prevetmed.2021.105266, PMID: 33517159

[64] Gradé JT Tabuti JRS Van Damme P. Four footed pharmacists: indications of self-medicating livestock in Karamoja, Uganda. Econ Bot. (2009) 63:29–42. doi: 10.1007/s12231-008-9058-z

[65] Ekwem D Morrison TA Reeve R Enright J Buza J Shirima G. Livestock movement informs the risk of disease spread in traditional production systems in East Africa. Sci Rep. (2021) 11:16375. doi: 10.1038/s41598-021-95706-z, PMID: 34385539 PMC8361167

